# Frameless Stereotaxy in Stereoelectroencephalography Using Intraoperative Computed Tomography

**DOI:** 10.3390/brainsci15020184

**Published:** 2025-02-12

**Authors:** Alexander Grote, Marko Gjorgjevski, Barbara Carl, Daniel Delev, Susanne Knake, Katja Menzler, Christopher Nimsky, Miriam H. A. Bopp

**Affiliations:** 1 Department of Neurosurgery, University Hospital Marburg, Philipps University Marburg, Baldingerstrasse, 35043 Marburg, Germanybarbara.carl@helios-gesundheit.de (B.C.); nimsky@med.uni-marburg.de (C.N.); 2 Department of Neurosurgery, Helios Dr. Horst Schmidt Kliniken, Ludwig-Erhard-Straße 100, 65199 Wiesbaden, Germany; 3 Department of Neurosurgery, Friedrich-Alexander University of Erlangen, Maximilianplatz 2, 91054 Erlangen, Germany; daniel.delev@uk-erlangen.de; 4 Epilepsy Center Hesse, Department for Neurology, University Hospital Marburg, Philipps University Marburg, Baldingerstrasse, 35043 Marburg, Germany; knake@med.uni-marburg.de (S.K.); katja.menzler@med.uni-marburg.de (K.M.); 5 Center for Mind, Brain and Behavior (CMBB), 35043 Marburg, Germany; 6 LOEWE-Research-Cluster for Advanced Medical Physics in Imaging and Therapy (ADMIT), Technische Hochschule Mittelhessen (THM), University of Applied Sciences, 35390 Giessen, Germany

**Keywords:** SEEG, invasive diagnostic, epilepsy surgery, accuracy, navigation

## Abstract

**Background:** Pharmacoresistant epilepsy affects approximately one-third of all epilepsy patients, and resective surgery may offer favorable outcomes for carefully selected patients with focal epilepsy. The accurate identification of the epileptogenic zone (EZ) is essential for successful surgery, particularly in cases where non-invasive diagnostics are inconclusive. Invasive diagnostics with stereoelectroencephalography (SEEG) offer a reliable approach to localizing the EZ, especially in MRI-negative cases. **Methods**: This retrospective study analyzed the data of 22 patients with pharmacoresistant epilepsy who underwent frameless stereotactic SEEG electrode implantation with automated CT-based registration between September 2016 and November 2024. For measuring accuracy, Euclidean distance, radial deviation, angular deviation, and depth deviation were calculated for each electrode. **Results:** A total of 153 depth electrodes were implanted, targeting various cortical regions. The median Euclidean distance at the entry point was 1.54 mm (IQR 1.31), with a radial deviation of 1.33 mm (IQR 1.32). At the target level, the median Euclidean distance was 2.61 mm (IQR 1.53), with a radial deviation of 1.67 mm (IQR 1.54) and depth deviation of 0.95 mm (IQR 2.43). Accuracy was not significantly affected by electrode order, anatomical location, skull thickness, or intracranial length. **Conclusions**: These findings demonstrate that frameless stereotactic SEEG electrode implantation is safe and feasible for identifying the EZ. The integration of automatic intraoperative CT-based registration ensures precision. While maintaining workflow efficiency, it achieves accuracy comparable to frame-based methods. Further studies with larger cohorts are warranted to validate these results and assess their impact on surgical outcomes.

## 1. Introduction

Around one-third of all epilepsy patients are pharmacoresistant, often presenting with temporal lobe epilepsy (TLE), and 20% show extratemporal seizure onsets [1,2,3,4,5]. For carefully selected patients with focal pharmacoresistant epilepsy, resective surgery is a well-established and promising treatment option, showing excellent outcomes especially in TLE patients [4,6], but posing a greater challenge in extratemporal lobe epilepsy [7,8,9]. However, one major prerequisite for resective surgery is the clear and precise approximation of the epileptogenic zone (EZ). Especially in cases in which non-invasive multimodal diagnostics fail to accurately define the EZ due to inconclusive radiological, clinical, semiological, and neuropsychological findings, further invasive diagnostics utilizing intracranial electroencephalography (iEEG) might be useful [10,11].

The current common practice for performing iEEG is the so-called stereo EEG (SEEG), which is based on the implantation of depth electrodes. Following anatomo-electro-clinical hypotheses, SEEG allows for the identification of seizure origin and propagation and a definition of the EZ. SEEG depth electrode implantation can generally be performed using frame-based stereotaxy or frameless navigation, with or without robotic assistance [12,13,14,15]. Accuracy is crucial in determining the risk of intracranial complications and the likelihood of successful EEG recordings and localization of the epileptogenic zone [15], independent of the applied technique. Clinical accuracy is a multifactorial parameter. In stereotactic and navigational applications, overall or clinical accuracy, which is most important to the surgeon, can be roughly divided into application accuracy and intraoperative accuracy, and the surgeon tremendously depends on accuracy in every single step of the procedure. Application accuracy itself can be divided into three domains as follows: imaging, technical, and registration accuracy [16,17,18]. Imaging accuracy mainly concerns the precise and optimized multimodal planning of trajectories incorporating target and risk structures, and the modality also ensures geometrical accuracy. The technical accuracy of the navigational systems depends, e.g., on the technique used (frame-based vs. frameless); the intrinsic accuracy of the systems itself; or the tracking technique used, which is nowadays considered to be less than 3 mm in frameless systems and even less in frame-based stereotactic systems, aiming for submillimeter accuracy [16,19]. Especially in frameless setups, registration accuracy mainly influences the application accuracy, offering user-dependent methods for landmark- and surface-based techniques with mean target registration errors of up to 5 mm [20,21,22] or automated intraoperative imaging-based techniques with higher registration accuracy and with target registration errors (TREs) of less than 1 mm [18,23,24,25,26]. While the domains mentioned above are related to the presurgical phase, intraoperative events hamper overall accuracy during the course of surgery. Navigational accuracy is known to decrease over time, which is related to, e.g., the attachment of drapes, incision, trepanation/drilling, and the duration of the surgery itself. These impact the spatial relationship between the patient’s head and the reference unit (reference array vs. frame), affecting the non-linear deformations of the brain, which might be a minor issue compared to craniotomy cases.

Several methods for measuring accuracy have been introduced recently, and these are inconsistently used across different studies, dealing with the accuracy of depth electrodes in epilepsy patients. The most prominent ones are the Euclidean distance at the entry and target points, which describe the three-dimensional deviation between the planned and detected trajectory endpoints. However, especially in the case of the target points, the radial and depth errors can be considered instead, as the depth error, incorporated in the Euclidean distance, is surgeon-dependent, thereby not reflecting the accuracy of the used approach [15]. In addition to the radial error, the angular deviation between the planned and detected trajectory can also be considered. Another study suggested reporting the directional errors instead to assess the systematic error in the stereotactic system [27].

Despite a potential loss of implantation accuracy when using frameless implantation techniques instead of frame-based methods, this study aims to evaluate the effect of utilizing automated intraoperative imaging-based techniques for patient registration, which are known to offer higher accuracy, on the implantation accuracy of SEEG depth electrodes.

## 2. Materials and Methods

### 2.1. Patient Cohort

This study analyzed the data of 22 patients with pharmacoresistant epilepsy who underwent implantation of SEEG electrodes using a frameless stereotactic approach between September 2016 and November 2024. All patients were pre-surgically assessed following a standard protocol, including clinical, radiological, neuropsychological, and non-invasive EEG diagnostics. In all cases, surgery for invasive diagnostics using SEEG depth electrodes was indicated following an interdisciplinary discussion (epileptology, neurosurgery, neuropsychology, and neuroradiology).

The local ethics committee at the University of Marburg granted ethics approval (No. 99/18) for prospectively collecting routine clinical and technical data during neurosurgical treatment in accordance with the Declaration of Helsinki. The committee also permitted the retrospective analysis of the collected data (RS 25/7). All patients provided informed consent.

### 2.2. Operating Room Equipment

All included patients underwent frameless navigation-supported surgery for invasive diagnostics using SEEG depth electrodes in a standardized setup. Therefore, the operating room was equipped with a mobile 32-slice intraoperative CT (iCT) system (AIRO^®^, Brainlab, Munich, Germany); a neuronavigation system (Curve Navigation, Brainlab, Munich, Germany; previously Curve CM, Brainlab, Munich, Germany) allowing for multimodal trajectory planning and navigation; the frameless stereotactic VarioGuide system (Brainlab, Munich, Germany); and VarioGuide compatible placement instruments for depth electrodes (Ad-Tech Medical Instruments Cooperation, Racine, WI, USA).

### 2.3. Preoperative Imaging and Planning for SEEG Placement

Following standard clinical diagnostic imaging, preoperatively, all patients underwent multimodal MRI imaging using a 3T MRI system (Tim Trio, Siemens, Erlangen, Germany) equipped with a 12-challe head matrix Rx-coil. The multimodal data set routinely included a 3D T1-weighted, 3D T2-weighted, 3D FLAIR (fluid-attenuated inversion recovery), time-of-flight (ToF) angiography, and a diffusion-weighted (DWI) single-shot echo planar imaging (EPI) data set with 30 non-colinear diffusion encoding gradients (b-values: 0 s/mm^2^, 1000 s/mm^2^) enabling the fiber tractography of the relevant major white matter tracts. If SEEG placement was considered to be closely related to the language-associated cortex, functional MRI (fMRI) data sets for the localization of Broca’s and Wernicke’s areas were assessed using tasks such as silent word generation, semantic decision, and passive listening.

In the first step, all required and available data sets were rigidly co-registered using the image fusion element (Brainlab, Munich, Germany). After the manual realignment of the data, depending on the localization of the suspected epileptogenic foci, various anatomical structures, such as the amygdala and hippocampus in TLE cases, were segmented automatically using the anatomical mapping element (Brainlab, Munich, Germany). In addition, if applicable, lesions and vascular risk structures were manually segmented using the smart brush element in MRI-positive cases (Brainlab, Munich, Germany). Depending on the covered region of the brain, major white matter tracts, such as the corticospinal tract, the arcuate fascicle, and the optic radiation, were visualized using the fiber tracking element (Brainlab, Munich, Germany), implementing a standard diffusion tensor imaging-based deterministic approach. Upon availability, fMRI data were analyzed using SPM8/SPM12, following standard protocol without normalization, revealing the activation clusters (family-wise error corrected) incorporated into the multimodal preoperative plan. Based on that preparatory work, the SEEG depth electrode locations were carefully planned using the trajectory element (Brainlab, Munich, Germany), which manually optimized the surgical trajectories for the maximized coverage of gray matter while minimizing the risk of vascular conflicts.

### 2.4. Intraoperative Workflow

Under general anesthesia, the patient’s head was secured in a radiolucent carbon head clamp (DORO, Black Forest Medical Group, Freiburg, Germany) using three metallic pins. Depending on the planned trajectories, the patient’s head was positioned in a supine, 45° tilted, lateral left, lateral right, or prone position; in the case a bilateral temporal implantation scheme was chosen, the first hemisphere was targeted. Afterward, the patient’s head was repositioned for the second hemisphere, following the same surgical procedure as in the first part of the surgery. Pin-related artifacts were less of an issue for the low-dose registration scans, while the full-dose control scan was performed at the end of the surgery, after the head clamp was detached. A radiolucent patient reference geometry was mounted at the head clamp’s left side to allow for navigation support. Three adhesive skin markers were attached to the patient’s head spread across the scan area of the registration scan to provide an estimate of the registration accuracy.

**Figure 1:**
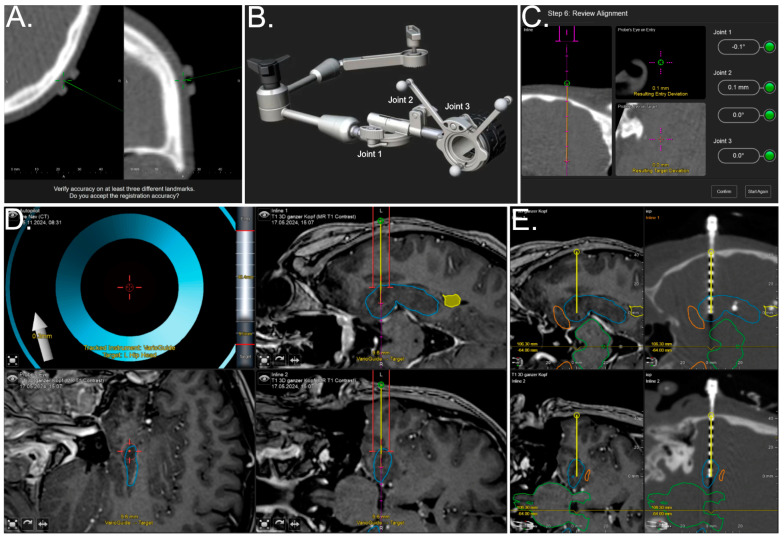
After automated iCT-based registration, the accuracy of registration is inspected and verified using skin-adhesive markers placed on the patient’s head within the scan region (**A**). Following the initialization of the VarioGuide with all joints set to zero (**B**), the VarioGuide is adjusted joint by joint to fit the preplanned trajectory (**C**). The navigational screen displays the alignment of the VarioGuide (red lines, projected into the navigational field using a virtual extension) around the planned trajectory (yellow), which is intended to be placed in the left hippocampal head outlined in blue (**D**). Intraoperative analysis of trajectory placement accuracy utilizing rigidly fused preoperative MRI (**left**) and iCT (**right**) data, with the planned electrode (yellow) visualized over the implanted electrode, indicating a high level of implantation accuracy (**E**).

Automatic intraoperative patient registration was performed by applying a sequential low-dose iCT scan (7.1 mA, 120 kV, 1.92 s exposure time, 1 mm reconstructed slice thickness, matrix size 512 × 512, 33.3 cm^2^ field of view) with a limited scan length of 6.2 cm resulting in a dose length product of 17.8 mGy × cm. The offset between the physical pointer’s tooltip in the divot of the three skin markers and its visual representation was used to assess the target registration error (TRE), see Figure 1A. After high patient registration accuracy was verified, the iCT registration scan and the multimodal preoperative planning data were rigidly co-registered to allow for immediate navigation support. Further details on the setup and iCT-based registration workflow can be found elsewhere [24].

For the accuracy checks, the entry points between all trajectories on the patient’s head were marked at the skin level. After draping and attaching a sterile patient reference array, the VarioGuide was equipped with a suitable guide disk attached to the head clamp on the right side. All trajectories were implanted consecutively. For each trajectory, the VarioGuide was initialized with all joints zeroed (Figure 1B). After a first rough alignment of the VarioGuide’s head above the entry point and locking for the VarioGuide arm, all three joints (disk joint: rotation; array holder: translation + rotation; VarioGuide holder: rotation) were sequentially adjusted within a range from −0.3° to + 0.3° and −0.3 mm to 0.3 mm, respectively, see Figure 1C.

After the adjustment of the VarioGuide with the insertion tube, the entry point at skin level was marked, followed by the skin incision and minimal dissection of the tissue to further move the insertion tube to skull level. The drill was adjusted accordingly to assess the instrument’s depth, followed by dural opening and monopolar coagulation. After measuring the instrument’s depth, the anchor bolt was inserted in accordance with the measured bone’s thickness, and a stylet was used to penetrate the tissue along a defined length. Finally, the depth electrode (Ad-Tech Medical Instruments Cooperation, Racine, WI, USA) was inserted, fixated at the anchor bolt, and secured with a plastic cover.

After the consecutive implantation of depth electrodes, a second iCT scan was performed to verify the electrodes’ spatial location and identify any immediate surgical complications (Figure 1E). In the case of bitemporal implantation, the second iCT scan was a low-dose registration scan after patient repositioning, followed by implantation as described above, concluding with a third iCT scan for final verification.

### 2.5. Analysis of Electrode Positioning Accuracy and Assessed Parameters

Based on the intraoperative CT at the end of the surgery, all placed electrodes were manually detected within the trajectory planning element (Brainlab, Munich, Germany) by matching a virtual representation of the electrodes with the metal artifacts in the iCT data. The similarity of planned and implanted electrode trajectories was analyzed after the co-registration of the verification iCT scan and the planning data. All trajectories were thereby described via the coordinates of the target point $TP=\left(x_{TP},y_{TP},z_{TP}\right)$ and the entry point $EP=\left(x_{EP},y_{EP},z_{EP}\right)$ at the skull’s outer surface. The trajectories can be described as straight lines $p:EP_{p}+\gamma \times P$ and $d:EP_{d}+\gamma \times D$, with $P=TP_{p}-EP_{p}$ and $D=TP_{d}-EP_{d}$.

Based on this, several parameters were calculated to evaluate the accuracy of electrode positioning by comparing the planned and detected electrodes as follows:

- Euclidean distance *ED_EP_* and *ED_TP_* between the planned and detected entry and target points, respectively, as follows:

$$ED_{EP}\left(EP_{p},EP_{d}\right)=\sqrt{{\left(x_{EP_{p}}-x_{EP_{d}}\right)}^{2}+{\left(y_{EP_{p}}-y_{EP_{d}}\right)}^{2}+{\left(z_{EP_{p}}-z_{EP_{d}}\right)}^{2}}$$

$$ED_{TP}\left(TP_{p},TP_{d}\right)=\sqrt{{\left(x_{TP_{p}}-x_{TP_{d}}\right)}^{2}+{\left(y_{TP_{p}}-y_{TP_{d}}\right)}^{2}+{\left(z_{TP_{p}}-z_{TP_{d}}\right)}^{2}}$$

- Angular deviation *AD_p,d_* as the angle between the two trajectory vectors *P* and *D*, as follows:

$$AD_{p,d}=\arccos\left(\frac{\left|P\times D\right|}{\left|P\right|\times \left|D\right|}\right)$$

- Radial deviation *RD* describes the distance between the intersection S of the detected trajectory and the normal plane of the planned trajectory at the level of the entry point and the detected entry point and at the level of the target point and the detected target point, respectively, as follows:

$$RD_{EP}=ED\left(S_{EP,\;}EP_{p}\right)\;mit\;S_{EP}=EP_{d}+\gamma^{\prime }\times D\;und\;(EP_{d}+\gamma^{\prime }\times D-EP_{p})\times P=0$$

$$RD_{TP}=ED\left(S_{TP,\;}TP_{p}\right)\;mit\;S_{TP}=EP_{d}+\gamma^{\prime \prime }\times D\;und\;(EP_{d}+\gamma^{\prime \prime }\times D-TP_{p})\times P=0$$

- Depth deviation D_TP_ at the target level describes the Euclidean distance between the calculated intersection *S_TP_* of the detected trajectory and the normal plane of the planned trajectory at the level of the target point

$$D_{TP}=ED\left(S_{TP,\;}TP_{d}\right)$$

In addition to the calculated accuracy parameters for each trajectory, additional information was assessed, including the position of the patient’s head (supine, prone, lateral left/right, 45° tilted left/right), the order of electrode placement, intracranial electrode length, bone thickness, muscle/tissue thickness, and time per electrode (ranging from setting up the VarioGuide for the trajectory to finishing the implantation of the electrode).

### 2.6. Statistical Analysis

Statistical Analysis was performed using the open source software jamovi from the jamovi project (Version 2.3.21, computer software, retrieved from https://www.jamovi.org, accessed on 1 December 2024) [28]. To analyze the impact of covariates, either a one-way ANOVA or Pearson correlation was used. If data showed no normal distribution according to the Shapiro–Wilk test, data were reported as the median with the corresponding interquartile range (IQR). Otherwise, the mean and standard deviation were utilized. The significance level was set as *p* < 0.05.

## 3. Results

### 3.1. Clinical and Demographic Information

This study included 22 patients (mean age: 36.94 ± 12.09 years; male/female: 13/9) undergoing surgery for invasive diagnostic using SEEG depth electrodes. Based on the non-invasive diagnostics, including surface EEG, imaging, and neuropsychological investigations, 14 patients presented with temporal lobe epilepsy. In some of these cases, iEEG was indicated to distinguish fast propagation from bilateral TLE. Seven patients showed frontal lobe epilepsy and one right parietal lobe epilepsy. Five patients were MRI-negative at the time of intervention. The remaining patients were MRI-positive, partially with non-specific lesions. For detailed patient information, see Table 1.

In total, 153 depth electrodes were implanted, 75 within the left and 78 within the right hemisphere. Of these, 52 depth electrodes targeted temporomesial structures, 13 were placed temporolaterally, 72 were implanted in the frontal region, 13 were parietal, and three were occipital. In ten cases with bilateral implantation, surgery was split into two phases, including patient repositioning and patient registration. A total of 50 electrodes were implanted in the lateral right, 48 in lateral left patient positioning, 40 electrodes in supine, 8 in prone, and 7 electrodes in supine, 45° right-tilted patient positioning. Details on patient positioning, the number of electrodes, and the electrode locations can be found in Table 2.

The median planned electrode length was 43 mm (IQR 13.5; min: 23.5 mm, max: 102.5 mm), with a tissue/muscle thickness of 9.0 mm (IQR 5.5; min: 4 mm, max: 25 mm) and a mean bone thickness of 5.5 mm (IQR 5.0; min: 2 mm, max: 14.5 mm).

Frameless navigation was facilitated with a mean initial TRE of 0.70 ± 0.30 mm. Time for implantation per depth electrode was 13.76 ± 4.75 min on average.

### 3.2. Accuracy Metrics

**Figure 2:**
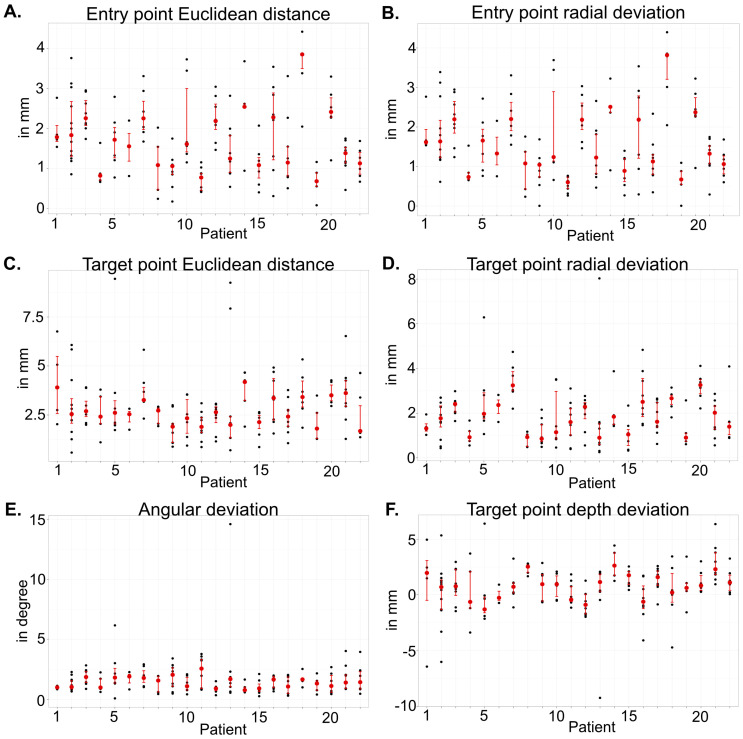
Case-wise accuracy parameter scatterplots for implanted electrodes per patient with the visualized median (red dot) and corresponding IQR (red): (**A**) Euclidean distance (1.54 mm, IQR 1.31) and (**B**) radial deviation (1.33 mm, IQR 1.32) at the entry point; (**C**) Euclidean distance (2.61 mm, IQR 1.53) and (**D**) radial deviation (1.67 mm, IQR 1.54) at the target point; (**E**) angular deviation (1.40°, IQR 1.11) between planned and implanted electrodes; and (**F**) depth deviation (0.95 mm, IQR 2.43) at the target point.

Figure 2 visualizes the accuracy metrics on a case-based level as follows: the median and IQR for the Euclidean distance and the radial deviation at entry level, the angular deviation between the planned and implanted electrode, and the Euclidean distance and radial and depth deviation at target level. Details on the metrics can be found in Table 3.

**Figure 3:**
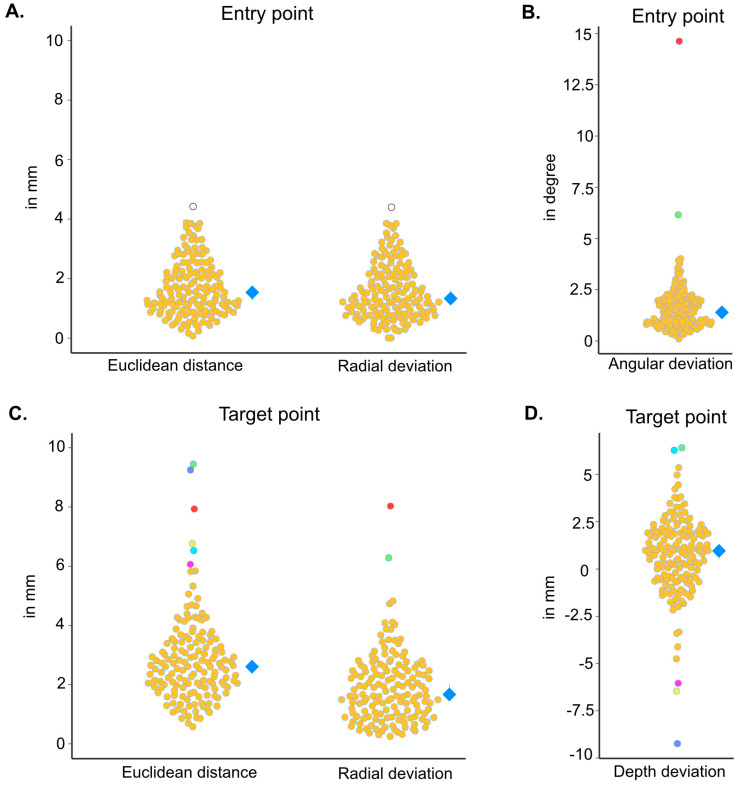
Overview of the accuracy metrics for all electrodes (orange) with the corresponding mean and IQR (blue diamond): (**A**) Euclidean distance and radial deviation at entry point, (**B**) angular deviation between planned and implanted electrodes, (**C**) Euclidean distance and radial deviation at the target point, and (**D**) depth deviation at the target level. The statistical outliers appearing in different accuracy domains are colored case-wise in white, green, dark blue, red, yellow, light blue, and pink, and these are outlined separately. Based on the IQR, outlier detection was performed to identify electrodes with the most significant error to identify possible sources of inaccuracy. For the Euclidean distance at the entry point (**A**), electrode p18/e3 was identified as an overall outlier with an error of 4.42 (case-wise median: 3.85 mm (IQR 0.37)), which was also accordingly seen in the radial deviation at the entry point (error: 4.40 mm, case-wise median 3.82 mm, IQR 0.65), see white circles in (**A**), showing a strong correlation (Pearson’s r = 0.983). (**B**–**D**) In the case of the angular deviation, five statistical outliers were detected as follows: p13/e9 (14.63°), p5/e6 (6.16°), p21/e3 (4.01°), and p22/e6 (3.94°), p11/e5 (3.76°). Regarding the accuracy metrics at target level in the case of the Euclidean distance, eight outliers were identified as follows: electrodes p5/e6 (9.45 mm), p13/e7 (9.25 mm), p13/e9 (7.93 mm), p1/e4 (6.76 mm), p21/e2 (6.52 mm), p2/e11 (6.07 mm), p2/e3 (5.84 mm), p7/e5 (5.83 mm); in case of the radial deviation (**C**), two outliers were detected as follows: p13/e9 (8.03 mm) (red circle), and p5/e6 (6.29 mm) (green circle). These match the cases with the largest angular deviation (**B**). Depth deviation (**D**) across all electrodes showed six statistical outliers for electrodes p13/e7 (−9.24 mm), p1/e4 (−6.46 mm), p5/e6 (6.42 mm), p21/e2 (6.37 mm), p2/e11 (−6.04 mm), and p18/e4 (−4.75 mm). The most prominent angular deviations (p13/e9 and p5/e6) resulted in corresponding large radial deviations, increasing the Euclidean distance at the target point. The second one was additionally affected by a relevant depth deviation (see (**B**–**D**), red (p13/e9) and green (p5/e5) dots). The other four most considerable discrepancies at the target level were mainly related to four outliers with high depth deviation (see (**B**–**D**), pink, light blue, yellow, and blue dots).

Across all patients and electrodes, the median Euclidean distance at the entry point was 1.54 mm (IQR 1.31), with a radial deviation at the entry level of 1.33 mm (IQR 1.32). The angular deviation between planned and detected electrodes showed a median of 1.40° (IQR 1.11). At the target level, a median Euclidean distance of 2.61 mm (IQR 1.53) was observed, with a median radial deviation of 1.67 mm (IQR 1.54) and a median depth deviation of 0.95 mm (IQR 2.43), see Figure 3.

Several parameters that potentially influence the implantation accuracy concerning the Euclidean distance and radial deviation at the target point were tested. Based on the analysis of 153 electrodes, the order of electrodes (*p* = 0.427; *p* = 0.346), temporal vs. extratemporal location (*p* = 0.442; *p* = 0.896), skull thickness (*p* = 0.349; *p* = 0.412), and intracranial length of the electrode (*p* = 0.211; *p* = 0.072) did not show a significant influence on electrode placement accuracy in this cohort. Three experienced senior neurosurgeons performed the surgeries. Inter-surgeon comparisons did not reveal any significant influence on the target accuracy parameters (*p* = 0.132; *p* = 0.116).

### 3.3. Surgical Complications

There were no surgical complications (intracranial hemorrhage, infection) among the reported cases on the intraoperative CT scan at the end of surgery or the postoperative MRI one day after surgery. In one case, the patient experienced pruritus without any signs of infection. Manipulation led to a loosening of one bone anchor, so this electrode was removed intermittently. In both cases with increased deviation (patients 5 and 13), inadequate positioning could be utilized for iEEG, resulting in a surgical recommendation for resection.

### 3.4. SEEG and Surgical Recommendations

Based on anatomo-electro-clinical hypotheses derived from non-invasive diagnostics, SEEG facilitated the identification of seizure origin and propagation and the delineation of the EZs in all cases, which is the primary goal of SEEG depth electrode implantation. SEEG promptly identified twelve patients as candidates for surgical resection (54.55%). In total, the EZ was located in 17 cases (77.27%). In the other five cases (22.73%), further invasive diagnostics using subdural electrodes were recommended to thoroughly map the eloquent cortex and delineate the cortical extent of the EZ prior to potential resective surgery. In five cases (22.73%), bilateral or multifocal epilepsy was diagnosed through SEEG, resulting in their exclusion from surgical recommendations.

## 4. Discussion

If standalone therapy with anti-seizure medication fails, resective surgery is a promising, well-established treatment option for carefully selected patients [1,2,3,4,5,6,7,8,9], if the EZ is clearly and precisely identifiable. However, in some cases, non-invasive multimodal diagnostics fail to accurately localize the EZ due to inconclusive radiological, clinical, semiological, and neuropsychological findings, or due to a potentially bilateral EZ. In these cases, invasive investigations, guided by hypotheses derived from non-invasive evaluations, become necessary [10,11]. These investigations typically involve the use of grid or depth electrodes.

Since Talairach and Bancaud introduced a new concept of the EZ [29,30], depth electrodes for SEEG have become an established procedure for invasive epilepsy diagnostics [31]. Compared to grid electrodes [30,32,33,34], depth electrodes are associated with lower complication rates. Accurate SEEG implantation is critical in ensuring sampling from the suspected EZ and minimizing risks, as safe implantation corridors between cerebrovascular risk structures are particularly narrow, especially when multiple electrodes are required [15,35]. Electrode malpositioning increases surgical risks and decreases the likelihood of identifying the EZ, reducing the chances of successful resection. Hence, carefully planning the hypothesis-driven preoperative trajectory is essential, with an emphasis on maximizing gray matter sampling while considering neurovascular risk. Incorporating knowledge regarding the accuracy of the utilized implantation method is crucial for ensuring safety and efficacy [15,36].

### 4.1. Different Techniques for SEEG Electrode Implantation

Several techniques for SEEG electrode placement are utilized, including frame-based stereotaxy and frameless navigation, with or without robotic assistance [12,13,14,15]. Frame-based stereotaxy is a well-established method for SEEG implantation and deep brain stimulation in general. This technique is known for providing high mechanical accuracy and demonstrating impressive precision in electrode placement, allowing for the implantation of depth electrodes that reach the desired target without neurovascular conflicts [13,15,32,37,38,39]. However, there are some drawbacks, such as potential patient discomfort; additional time required for frame setup; overall time consumption; limited access to the surgical field; and, depending on the accessibility of the surgical field, a restricted ability to define new trajectories during surgery; as well as the necessity for specialized equipment [37]. Despite the benefits of the frame-based approach, particularly regarding its purported superiority in accuracy compared to frameless techniques, frameless stereotactic methods are increasingly being adopted nowadays. Based on limited data for SEEG, but mainly for biopsy cases, frameless approaches could still offer acceptable accuracy and safety [32,37,40,41] with better real-time visualization of trajectories in parallel [32]. Robotic applications were recently introduced to the field of functional neurosurgery, emerging as safe, accurate, and time-saving methods for depth electrode implantation. The most used systems, Neuromate^®^ and ROSA^®^, allow their application in a frame-based or frameless manner. Their accuracy, even though reducing user bias, depends on the accuracy of the underlying technique (frame-based vs. frameless) [32,42].

### 4.2. Quantifying Accuracy

Various measures of accuracy have been introduced, but they have been reported inconsistently across different studies analyzing the accuracy of depth electrodes used in epilepsy and deep brain stimulation. The Euclidean distance at the entry and target points is frequently reported to quantify implantation accuracy according to the planned trajectory. Additionally, some studies investigate the lateral deviation at the entry and target points, without considering depth deviations along the trajectory. Depth deviations are less significant for the entry point on the skull’s outer surface. When comparing Euclidean distance and lateral deviation, both depth and lateral deviation significantly influence the Euclidean distance for the target point. In cases of SEEG, depth is controlled by the surgeon and is independent of the technique used; thus, it can be somewhat variable. To better assess the accuracy of the method itself, some authors recommend employing lateral deviation instead of the Euclidean distance at the target point as a measure of accuracy. Moreover, the angular deviation between the planned trajectory and the implanted electrode can also be considered, as it is closely related to the lateral deviation at the target point [15].

### 4.3. Accuracy of SEEG Techniques

As stated in a recent meta-analysis, there is heterogeneity in reporting accuracy across different studies, particularly regarding accuracy and the statistical descriptive parameters (e.g., mean/median), which may lead to inaccuracies when comparing studies [15]. Some studies report only the Euclidean distance, while others focus on lateral deviation.

Utilizing the Euclidean distance in a recent comparable study with a different operating room and procedural setup, a mean error was observed at the entry point of 2.7 ± 2.2 mm and at the target point of 4.6 ± 2.1 mm [43]. A recent meta-analysis [15] showed a mixed Euclidean distance/lateral deviation mean error at the entry point of 2.45 mm (95% CI: 0.39–4.51) and at the target point of 2.89 mm (95% CI: 2.34–3.44) was reported. Another study investigating frameless navigation with intraoperative MRI showed an error at the entry point of 1.4 mm ± 1.2 mm and at the target point of 3.2 ± 2.2 mm, ranging from 0 to 8.6 mm [44]. In two other studies with small sample sizes, only target deviations of 3.0 ± 1.9 mm [41] and 3.5 mm (95% CI: 2.9–3.9) ranging from 1.2 to 13.7 mm were detected [45]. In a recent study, also utilizing the VarioGuide in a frameless setup and relying on fiducial-based registration, a mean Euclidean distance of 4.2 ± 2.47 mm at the entry point and 4.06 ± 2.5 mm at the target point was reported [46]. Relying on a landmark-based registration utilizing bony features, a mean Euclidean distance at the entry point of 3.64 ± 1.78 mm and of 2.96 ±1.49 mm at the target point was reached [40], compared to a study using VarioGuide in a frameless setup and relying on surface-based registration, which reached a mean Euclidean distance of 3.2 ± 2.4 mm for the entry point and 2.7 ± 2.0 mm for the target point [47]. The results of this study, using an automatic iCT-based registration for frameless stereotaxy and utilizing the Euclidean distance and radial deviation at the entry point (median: 1.54 mm; IQR 1.31 and 1.33 mm, IQR 1.32) and at the target point (median: 2.61 mm; IQR 1.53 and 1.67 mm, IQR 1.54), favorably compare with the reported errors, showing, in part, an even higher accuracy, despite the frameless setup.

In the case of traditional frame-based implantation, which represents the state-of-the-art implantation of depth electrodes for deep brain stimulation and SEEG [44], expecting higher accuracy, one study reported a median error at the target point of 2.02 mm (IQR 1.59) [13]. In a recent study investigating a frame-based approach with modern visualization systems, a mean Euclidean distance at the entry point of 0.6 ± 0.5 mm and at the target point of 1.5 ± 0.8 mm was achieved, with an additional report on the lateral deviation at the target level of 1.1 ± 0.7 mm, showing the expected higher precision in the frame-based setup [32]. Another study on frame-based SEEG revealed a mean Euclidean distance of 1.5 ± 0.6 mm at the entry point and 1.5 ± 0.8 mm at the target point [48]. Within the above-mentioned meta-analysis, in the case of the frame-based setup, mixed Euclidean distances/lateral deviations of 1.43 mm (95% CI: 1.35–1.51) and 1.93 mm (95% CI: 1.05–2.81) at the entry point were revealed. Another study reporting on the use of a frame-based approach for SEEG implantation reached a median Euclidean distance of 1.54 mm (IQR 1.36) at the entry point and 2.93 mm (IQR 2.22) at the target point [34], which is consistent with the results achieved by the frameless approach presented in this study. Closely looking at accuracy measures in the frame-based setup, there are several reports showing higher inaccuracies up to a mean Euclidean distance of 7–8 mm at the target point. These reports also show a markedly broader error range, implying that the frame-based setup is also prone to errors [39,49].

### 4.4. Factors Influencing Accuracy

The safety and potential advantages of image-guided surgery, in either frame-based or frameless setups, crucially depend on the accuracy and precision of linking the physical patient space with the image space, as well as transferring the multimodal preoperatively generated data and surgical plan to the intraoperative surgical site [16,17,50]. In the current literature, the term “accuracy” is not consistently defined or used. Clinical or overall accuracy, particularly in relation to the surgeon, is a combination of application and intraoperative accuracy. Application accuracy, as proposed, can be divided into imaging, technical, and registration accuracy, while intraoperative accuracy largely depends on events occurring during the procedure [17]. Marginal gains in accuracy at every step contribute to the overall accuracy, which is crucial for both the surgeon and patient safety.

*Imaging accuracy* depends on the imaging modality used, such as CT or MRI, as well as the imaging parameters (e.g., resolution, contrast, parallelization) [51]. CT exhibits fewer geometric distortions compared to MRI, which allows for the more precise spatial registration of the patient to the image [52]. In brain surgery, MRI is the preferred modality, offering the best tissue contrast within the brain for optimized trajectory planning to reach the target while maximizing distance to risk structures. When both modalities are available, electrode localization is preferably performed using CT data rather than MRI data [53], even though it is comparably feasible with both [54,55,56]. In such cases, CT/MRI image fusion is conducted. However, non-linear geometric distortions within the MRI data can lead to suboptimal image fusion results, introducing spatial inaccuracies into the overall accuracy chain [57]. Image quality, the quality of multimodal image fusion, and a consideration of non-linear spatial distortions therefore impact the spatial accuracy of electrode planning (based on MRI), placement (most often relying on CT-image-based registration), but also of the electrode detection for the accuracy analysis [35].

*Technical accuracy* refers to the intrinsic accuracy of the navigational system in use, whether frameless or frame-based; the tracking technology utilized in the frameless setup; and the geometrical integrity of the tracked instruments employed (e.g., reference array, pointer) [58]. Currently, the technical accuracy of frameless stereotaxy is shown to be less than 3 mm, with an even higher accuracy in the frame-based setup [16], underpinning the generally higher electrode placement accuracy achieved when using the frame-based technique. The availability of intraoperative imaging provides a timely visual inspection of accuracy, enabling the user to update the navigation if needed, while also allowing for the identification of the potential causes of deviations. For instance, in this study, patient no. 18 exhibited consistently poorer accuracy at the entry point (Euclidean distance: median 3.85 mm (IQR 0.37); radial deviation: median 3.82 mm (IQR 0.65)), with a comparable directional shift observed in all electrode entry points, as seen at the end of the surgery, after performing an iCT scan. Immediately examining the navigational equipment, a slight tilting of one reference pin of the sterile reference array was noted, indicating a divergent geometry compared to the non-sterile one used for patient registration, which may explain the observed homogeneous shift and inaccuracy across all implanted electrodes in that case. To address this issue, a visual inspection of the geometrical integrity of the navigation equipment is necessary before surgery.

*Registration accuracy* is recognized as one of the primary factors influencing application accuracy [20,22,23], and various approaches are available [20]. In the frameless setup, patient registration is typically conducted using either landmark-based or surface-based registration techniques. In the landmark-based method, artificial markers are attached to the patient’s head before preoperative imaging, and these are examined intraoperatively, following patient positioning and head fixation for a paired point registration procedure [16]. This method has shown considerably varying TREs ranging from 1.8 to 5.0 mm [20,21], which depend on the number, position, and spatial arrangement of the markers; skin shifts during image acquisition and following head fixation, as well as the user-dependent registration procedure [16,18,59,60,61]. Surface-based techniques combine anatomical landmarks with laser surface matching, which is somewhat easier to use clinically for patient registration since it does not require explicit preoperative imaging with adhesive skin markers, although it is not suitable for use in a prone position. Nonetheless, the literature reports indicate that registration accuracy is even poorer when using this method compared to the landmark-based approach, with TREs of 5.3 mm, which heavily depends on the imaging modality and quality [22]. The introduction of intraoperative imaging has facilitated automatic and, therefore, user-independent registration procedures, significantly reducing the initial registration error with reported TREs of less than 1 mm [18,23,24,25,62]. This significantly diminishes the error introduced by one of the major contributing factors, initial patient registration, and enhances overall clinical accuracy compared to user-dependent approaches, which might also serve as one of the main contributing factors for SEEG depth electrode placement accuracy. This finding is also reflected in our study. When comparing this study, which employed automatic iCT-based registration and the use of the VarioGuide in a frameless setup, with studies using the VarioGuide but relying on landmark- or surface-based registration methods, the accuracy measures for SEEG electrode implantation in the presented setup are superior (e.g., Euclidean distance at the following target points: 2.61 mm vs. 4.06 mm vs. 3.2 mm), as detailed in the preceding paragraph, underpinning the importance of high registration accuracy as a prerequisite for high implantation accuracy.

Several factors compromise *intraoperative accuracy* following the registration procedure. Traditionally, in craniotomy cases, non-linear deformations of the brain during surgery—due to factors such as loss of cerebrospinal fluid, swelling, tumor mass resection, retractors, and gravitational effects—can impair accuracy [18,50,63]. However, their impact may be less significant in SEEG cases. Gravitational effects already arise during patient positioning; if the intraoperative positioning of the patient’s head changes from its preoperative position during MRI data acquisition, these gravitational effects lead to intracranial shifts, resulting in inaccuracies in the mapping images and the representation of intracranial structures in the physical patient space [64]. In the case of SEEG electrode placement, especially when only small corridors for safe electrode placement are available, this shift could significantly affect patient safety as the intracranial shifting of the brain cannot be compensated for by the latest registration techniques. Therefore, it may be worth considering acquiring preoperative MRI data with the patient’s head positioned accordingly. However, this could potentially reduce patient comfort during the imaging procedure and is not applicable in bilateral cases involving intraoperative patient repositioning. Previous studies on navigational accuracy have consistently reported a decrease in accuracy during surgery, alongside evident intracranial brain shifts, primarily related to the positional shift of the patient’s head concerning the reference array. This may be linked to intraoperative factors such as draping, the interchange between non-sterile and sterile reference arrays and their geometric integrity, the handling of navigational equipment, skin incision, drilling, and/or the duration of surgery [16,20,50]. Additionally, the choice of head immobilization devices might also contribute to this, leading to a slight decrease in overall accuracy as a baseline for electrode implantation accuracy. In frameless situations, a three-point head clamp is generally employed, while frame-based systems usually utilize a four-point head fixation, which can offer greater temporal stability. This underscores the need to verify navigational accuracy during surgical procedures, for instance, by using additional points on the patient’s head that are recorded immediately after the registration for verification purposes. In the specific instance of using a guiding device such as the VarioGuide, all joints must be reset to zero before each trajectory to ensure the correct initialization of the guiding device. In the case of SEEG, various procedure-related factors affect implantation accuracy, including drilling errors due to instabilities or slipping, the mechanical properties of the electrodes, the structural and biomechanical characteristics of the penetrated intracranial tissue, or the surgical techniques used (e.g., inserting the electrode with or without a stylet).

### 4.5. Complications

Various studies have already demonstrated safety and a lower complication rate than implantation using subdural grid electrodes [30,34], with a recent systematic review closely evaluating SEEG-related complications [38]. Pooling data from 30 studies reporting 121 complications showed a prevalence of 1.3% (95% CI: 0.9–1.7). Complications most commonly included intracranial hemorrhages (pooled prevalence: 1%) and infections (pooled prevalence: 0.8%), as well as technical complications (malfunction and malpositioning) with a pooled prevalence of 0.6% [38]. Single studies reported complication rates of 4.0% [65], 4.2% [30], and 2.4% [13] for hemorrhage and infection. In another study, a major complication rate of 1.52% and a minor complication rate of 2.09% were observed [66]. In this study, no surgical complications such as hemorrhage or infection were seen, which is comparable to the findings of other studies [43,44]. In one case, an electrode was accidentally removed. Technical complications regarding suboptimal positioning were seen in two cases (1.31%), and these were identified without any need for repositioning, as they could still be utilized for iEEG.

### 4.6. Study Strengths and Limitations

One of the strengths of this method is the robust number of implanted electrodes, which provide comprehensive data for statistical analyses. To the best of our knowledge, we report the first results using automatic intraoperative CT-based registration, smoothly integrated into the surgical workflow in combination with a frameless implantation device.

However, adopting the proposed approach or the other recently suggested methods for global SEEG electrode placement may present challenges and incur additional costs for specialized equipment. The outlined workflow, which incorporates navigation and intraoperative imaging, is not limited to SEEG electrode placement; it can also be applied to other neurosurgical procedures [23,24] and various disciplines, promoting shared use and cost-efficiency. Nonetheless, regardless of the availability of equipment and the surgical process discussed, many factors can be addressed and considered to enhance accuracy and minimize the loss of precision during surgery, ensuring precise electrode placement for patient safety and the effective identification of the EZ.

Besides its retrospective character, monocentric design, and inclusion of only one single implantation technique, the small sample size of 22 patients represents a significant limitation. Although nearly 160 electrodes were used for the statistical analysis of accuracy and the sample size is comparable to those of other recently published studies on SEEG depth electrode implantation (e.g., [32,43,44,46]), the statistical power remains limited, which hinders the generalizability of the results. Consequently, conclusions drawn from smaller cohorts should be interpreted with caution. Realizing the manifoldness of factors potentially influencing accuracy, to different degrees as reported in the recent literature, not all were considered in this study (e.g., the angulation between the trajectory and skull surface was reported to positively correlate with the radial deviation at the entry point [67]), and these should be more comprehensively analyzed in further analyses, while also pooling data from different centers and techniques.

Nevertheless, this study offers valuable insights into the accuracy of SEEG depth electrode placement and adds to the growing body of literature in this field, providing a foundation for future research.

## 5. Conclusions

This study highlights the feasibility and accuracy of using automatic intraoperative CT-based registration for frameless SEEG electrode implantation. The results demonstrate that this approach achieves accuracy comparable to the traditional frame-based methods while integrating seamlessly into the surgical workflow. By minimizing user-dependent variability in registration and enhancing accuracy through improved trajectory planning, this method offers a safe and reliable alternative for invasive epilepsy diagnostics. The findings also underscore the importance of optimizing each step of the surgical process, from imaging and registration to electrode placement, to ensure patient safety and the successful identification of the epileptogenic zone. Future studies with larger patient cohorts and prospective designs are needed to further validate these findings and explore their implications for surgical outcomes.

## Figures and Tables

**Table 1:** Patient characteristics.

Patient No.	Age	Sex	Epileptogenic Zone (Non-Invasive Diagnostics)	MRI Assessment
1	54.75	M	TLE, right	Multiple small and non-specific intracranial post-contusion defects
2	39.59	M	FLE, bilateral	-
3	37.62	M	PLE, right	-
4	42.37	F	TLE, left	T2/FLAIR hyperintense left insular lesion, left hippocampal sclerosis
5	16.45	F	FLE, left	Post-ischemic lesion left frontal
6	24.77	M	TLE, left vs. bilateral	Left hippocampal sclerosis
7	54.06	M	FLE, left	Left frontal heterotopia
8	33.30	F	TLE, left	Left hippocampal sclerosis
9	42.52	M	FLE, right	-
10	55.04	M	TLE, bilateral	-
11	29.39	M	TLE, left vs. bilateral	Left temporal ganglioglioma
12	23.10	M	FLE left	Dysplasia frontal left
13	30.81	F	FLE, left	-
14	45.98	M	FLE, right	Right frontomesial FCD
15	43.08	M	TLE, bilateral	Left hippocampal sclerosis
16	32.31	M	TLE, right	Right hippocampal sclerosis
17	43.68	F	TLE, bilateral	Left hippocampal sclerosis/DNET
18	26.60	F	TLE, right	Right hippocampal sclerosis
19	57.52	F	TLE, bilateral	Right (and developing left) hippocampal sclerosis
20	33.64	M	TLE, right	Right temporolateral lesion
21	16.68	F	TLE, right	Right temporal FCD
22	29.35	F	TLE, right	Right hippocampal sclerosis, Bilateral parietal nodular heterotopia

MRI: magnetic resonance imaging; FLAIR: fluid attenuated inversion recovery; DNET: dysembryoplastic neuroepithelial tumor; FCD: focal cortical dysplasia; FLE: frontal lobe epilepsy; PLE: parietal lobe epilepsy; TLE: temporal lobe epilepsy.

**Table 2:** Surgical plan and procedure.

Patient No.	Patient Positioning	Number of SEEG Depth Electrodes	Electrode Localizations
1	Lateral left	4	Right: temporomesial (3), frontal (1)
2	Lateral right, lateral left	12	Left: frontal (5), temporomesial (1), parietal (1); right: frontal (3), temporomesial (2)
3	Prone	8	Left: parietal (3), occipital (2); right: parietal (2), occipital (1)
4	Lateral right	5	Left: frontal (2), temporomesial (3)
5	Supine, 45° tilted to right	7	Left: frontal (4), temporomesial (1); right: frontal (2)
6	Supine	3	Left: temporomesial (2); right: temporomesial (1)
7	Lateral right	7	Left: frontal (4), temporomesial (1); right: frontal (2)
8	Lateral right	5	Left: parietal (3), temporolateral (2)
9	Supine	9	Left: frontal (1); right: frontal (8)
10	Lateral left, lateral right	6	Left: temporomesial (2); right: temporomesial (2), temporolateral (2)
11	Lateral right, lateral left	9	Left: temporomesial (3), temporolateral (3); right: temporomesial (3)
12	Supine	8	Left: frontal (6); right: frontal (2)
13	Supine	9	Left: frontal (6); right: frontal (3)
14	Supine	5	Left: frontal (1); right: frontal (4)
15	Lateral right, lateral left	7	Left: frontal (2), temporomesial (3); right: temporomesial (2)
16	Lateral left, lateral right	8	Left: frontal (1), temporomesial (1); right: frontal (4), temporomesial (1), temporolateral (1)
17	Lateral left, lateral right	9	Left: frontal (1), temporomesial (3); right: frontal (1), temporomesial (3), parietal (1)
18	Lateral left	6	Right: frontal (3), temporomesial (2), parietal (1)
19	Lateral left, lateral right	5	Left: temporomesial (2); right: temporomesial (2), temporolateral (1)
20	Lateral left, lateral right	6	Left: temporomesial (2); right: temporomesial (3), temporolateral (1)
21	Supine	9	Left: frontal (1); right: frontal (5), temporolateral (3)
22	Lateral right, lateral left	6	Left: temporomesial (2), parietal (1); right: temporomesial (2), parietal (1)

**Table 3:** Case-wise accuracy metrics reported as the median (IQR) in millimeters (Euclidean distance, radial and depth deviation) or degrees (angular deviation).

Patient No.	Entry Point Eucl. Distance	Entry Point Radial Deviation	Angular Deviation	Target Point Eucl. Distance	Target Point Radial Deviation	Target Point Depth Deviation
1	1.78 (0.40)	1.62 (0.37)	1.01 (0.25)	3.91 (2.91)	1.32 (0.33)	1.97 (3.59)
2	1.83 (1.37)	1.63 (0.95)	1.05 (0.68)	2.54 (1.25)	1.77 (0.95)	0.70 (2.67)
3	2.25 (0.63)	2.20 (0.80)	1.87 (0.93)	2.69 (0.79)	2.41 (0.44)	0.77 (2.35)
4	0.82 (0.17)	0.73 (0.17)	1.00 (0.84)	2.42 (1.39)	0.92 (0.44)	−0.64 (3.30)
5	1.72 (0.76)	1.66 (0.83)	1.82 (1.21)	2.61 (1.19)	1.97 (1.16)	−1.32 (1.41)
6	1.55 (0.70)	1.33 (0.70)	1.91 (0.71)	2.54 (0.54)	2.37 (0.61)	−0.28 (0.83)
7	2.25 (0.71)	2.20 (0.72)	1.79 (0.96)	3.27 (0.70)	3.25 (0.85)	0.72 (0.91)
8	1.08 (1.08)	1.08 (0.95)	1.58 (1.30)	2.73 (0.80)	0.93 (0.54)	2.53 (0.70)
9	1.06 (0.25)	1.04 (0.34)	2.05 (1.53)	1.90 (0.96)	0.86 (0.77)	0.96 (2.40)
10	1.61 (1.59)	1.24 (1.76)	1.11 (1.05)	2.33 (1.72)	1.15 (2.13)	0.95 (1.85)
11	0.77 (0.43)	0.60 (0.29)	2.56 (2.38)	1.89 (0.77)	1.60 (1.20)	−0.45 (1.39)
12	2.19 (0.64)	2.18 (0.67)	0.92 (0.26)	2.64 (0.81)	2.29 (0.72)	−0.91 (1.79)
13	1.24 (0.93)	1.23 (1.00)	1.70 (0.80)	2.00 (1.09)	0.90 (0.94)	1.15 (1.60)
14	2.54 (0.08)	2.51 (0.16)	0.79 (0.30)	4.19 (1.04)	1.84 (0.41)	2.63 (2.04)
15	1.08 (0.52)	0.89 (0.59)	0.93 (0.63)	2.14 (0.67)	1.05 (0.71)	1.76 (1.18)
16	2.28 (1.68)	2.19 (1.59)	1.67 (0.95)	3.37 (2.13)	2.51 (1.71)	−0.63 (2.05)
17	1.14 (0.74)	1.13 (0.50)	1.08 (1.37)	2.42 (0.85)	1.62 (1.10)	1.59 (1.29)
18	3.85 (0.37)	3.82 (0.65)	1.67 (0.17)	3.42 (1.33)	2.67 (0.41)	0.20 (2.58)
19	0.68 (0.34)	0.67 (0.34)	1.33 (0.77)	1.80 (1.32)	0.90 (0.36)	0.62 (0.72)
20	2.41 (0.49)	2.37 (0.47)	1.13 (1.40)	3.51 (0.81)	3.26 (0.64)	0.83 (1.34)
21	1.38 (0.39)	1.33 (0.46)	1.41 (1.12)	3.62 (1.32)	2.02 (1.04)	2.30 (1.98)
22	1.12 (0.54)	1.06 (0.46)	1.44 (1.43)	1.68 (1.37)	1.40 (0.54)	1.10 (1.37)

## Data Availability

The data in this study are available upon request from the corresponding author. The data are not publicly available due to privacy restrictions.
